# Teaching Immunology as a Science and Field of Medicine

**DOI:** 10.3389/fimmu.2014.00114

**Published:** 2014-03-20

**Authors:** Michelle Letarte, Wendy Tamminen

**Affiliations:** ^1^Department of Immunology, University of Toronto, Toronto, ON, Canada

**Keywords:** clinical immunology, teaching materials, health and disease, translational medical research, education, medical

Forty years after the publication of his first edition of Immunology, Dr. Joseph A. Bellanti has produced a fully revised and state-of-the-art fourth edition. The book has three main sections: (I) Principles of Immunology (Chapters 1–10); (II) Mechanisms of Response (Chapters 11–15); and (III) Clinical Applications (Chapters 16–25). The first section provides an initial focus on the fundamental concepts of immunity and the cellular and molecular players that form its basis. The second section then depicts the immune response in action against pathogenic threats, addressing physiological processes that protect the host, but that may also be evaded or may lead to immunopathology. The final section presents chapters with a more purely clinical focus, dealing in turn with all the major diseases and conditions related to immune function. All three sections are written with the intent of serving readers with varying backgrounds in immunology, from the complete novice to those looking for the latest consensus view on various topics in this always rapidly changing field.

Covering the realm of immunology in 25 or so chapters is the general goal of any of a number of standard immunology textbooks available, and most will likely have similar content presented in similar order. However, what distinguishes Immunology IV is the consistently high level of clinical integration found throughout the book, even in the first two sections, which do not cover “clinical topics” *per se*. Many textbooks make brief, sporadic clinical connections within non-clinical chapters, and sometimes refer to further coverage elsewhere; however, in Immunology IV there is a sense of ongoing immersion in the medical aspects of the material that goes beyond sporadic digression. At times this approach is less systematic but it is consistently engaging and effective for the reader. Another key feature that firmly establishes the clinical focus of the book is the inclusion of clinical case studies throughout. Each chapter begins with a relevant case that introduces the topic, and after full consideration of cellular and molecular mechanisms involved at both local and systemic levels, concludes with an explanation that touches on diagnosis, therapy, and prevention as applicable. In at least one example there is even a historical perspective; many books refer to or describe the experience of the late David Vetter, known internationally as “The Boy in the Bubble” both before and after his death at age 12 years from severe combined immunodeficiency, however, it is both powerful and poignant to see his story presented as a case study.

The needs of the medically oriented reader are also well-served by stand-alone chapters dedicated to some topics that are sometimes not included in other books, or covered only as relatively brief subtopics. So the reader will find full chapters on complement, inflammation, cytokines/chemokines, immunogenetics, vaccines, and lymphoproliferative disorders, as well as separate chapters describing immune responses to bacteria, viruses, parasites, and fungi. In addition, the middle section of the book leads with a chapter on clinical immunomodulation that describes general and specific approaches to boosting or reining in immune responses via the use of immunopotentiators or immunosuppressants, respectively. Finally, the third section of the book includes a comprehensive chapter on the laboratory diagnosis of diseases mediated by dysfunction of innate and adaptive components of the immune system. This practical chapter allows readers to have basic information about immune function assays in one convenient place for reference.

The book itself is very well-illustrated in full color and immune cell subsets and key molecules (summarized in a glossary) are clearly recognizable throughout. On the first page of each chapter, specific learning objectives are clearly stated, while key points are summarized at the end of the chapter to highlight the basic science material in a clinical context. “Study Questions/Critical Thinking” provided in each chapter will stimulate the inquisitive student. Highlight boxes appear in each chapter to deepen the discussion and further engage the readers. A fully searchable bibliography provides the key references for each chapter. The book also has three useful Appendices including abbreviations, lists of the major characteristics of common cytokines and chemokines, and the common CD molecules. Free 2-year access to the whole online book along with continuous updates is provided with purchase of the paper copy. The online content includes multilevel interactive teaching tools that guide the reader to the right answers and enrich the learning experience, as well as 800 color illustrations that allow a visualization of complex normal and pathophysiologic processes associated with basic and clinical immunological reactions. Computer generated animations provide a dynamic view of immune cells, their interactions with key molecules (cytokines, chemokines, and growth factors) and their production of effector molecules. Although the animation videos are excellent, each one appears several times and one would wish for more diversity.

“Immunology IV” provides a comprehensive survey of Human Immunology in Health and Disease. It is aimed at students of Clinical Immunology, whether they are scientists, medical students, or clinicians wanting to refresh their knowledge. It is a great textbook and the inclusion of clinical cases in each chapter should keep the reader highly engaged. The content is comprehensive in terms of teaching the basic knowledge needed to understand the mechanisms causing a vast array of immunological diseases. The level of detail in the first overview chapter may be somewhat daunting to the neophyte, but sets the stage for what is to come and this chapter could serve as an excellent review chapter to return to repeatedly throughout the learning process.

Overall, this book is extremely well-designed with the use of numerous teaching tools. We strongly recommend this book and will encourage its international distribution. We hope that it will serve as a reference textbook and guide for both teachers and students in many parts of the world, including developing countries.

